# Maintenance of Postharvest Quality and Reactive Oxygen Species Homeostasis of Pitaya Fruit by Essential Oil *p*-Anisaldehyde Treatment

**DOI:** 10.3390/foods10102434

**Published:** 2021-10-13

**Authors:** Yanmei Xu, Zhijun Cai, Liangjie Ba, Yonghua Qin, Xinguo Su, Donglan Luo, Wei Shan, Jianfei Kuang, Wangjin Lu, Liling Li, Jianye Chen, Yating Zhao

**Affiliations:** 1 State Key Laboratory for Conservation and Utilization of Subtropical Agro-Bioresources, Guangdong Provincial Key Laboratory of Postharvest Science of Fruits and Vegetables, Engineering Research Center of Southern Horticultural Products Preservation, Ministry of Education, South China Agricultural University, Guangzhou 510642, China; yljddcr123@163.com (Y.X.); qinyh@scau.edu.cn (Y.Q.); shanwei@scau.edu.cn (W.S.); jfkuang@scau.edu.cn (J.K.); wjlu@scau.edu.cn (W.L.); Meilingen@163.com (L.L.); chenjianye@scau.edu.cn (J.C.); 2 College of Food and Drug, Liaoning Agricultural Technical College, Yingkou 115009, China; 3 School of Food and Pharmaceutical Engineering, Guizhou Engineering Research Center for Fruit Processing, Guiyang University, Guiyang 550003, China; baliangjie@163.com (L.B.); luodonglan1991@163.com (D.L.); 4 Guangdong AIB Polytechnic, Guangzhou 510507, China; suxg@gdaib.edu.cn

**Keywords:** pitaya fruit, *p*-Anisaldehyde, quality, reactive oxygen species (ROS), AsA-GSH cycle, antioxidant activity

## Abstract

The performance of *p*-Anisaldehyde (PAA) for preserving pitaya fruit quality and the underpinning regulatory mechanism were investigated in this study. Results showed that PAA treatment significantly reduced fruit decay, weight loss and loss of firmness, and maintained higher content of total soluble solids, betacyanins, betaxanthins, total phenolics and flavonoids in postharvest pitaya fruits. Compared with control, the increase in hydrogen peroxide (H_2_O_2_) content and superoxide anion (O_2_^•−^) production was inhibited in fruit treated with PAA. Meanwhile, PAA significantly improved the activity of antioxidant enzymes superoxide dismutase (SOD), peroxidase (POD) and catalase (CAT). Moreover, PAA-treated pitaya fruit maintained higher ascorbic acid (AsA) and reduced-glutathione (GSH) content but lower dehydroascorbate (DHA) and oxidized glutathione (GSSG) content, thus sustaining higher ratio of AsA/DHA and GSH/GSSG. In addition, activities of ascorbate peroxidase (APX), glutathione reductase (GR), monodehydroascorbate reductase (MDHAR) and dehydrogenation ascorbic acid reductase (DHAR), as well as the expression of *HpSOD*, *HpPOD*, *HpCAT*, *HpAPX*, *HpGR*, *HpDHAR* and *HpMDHAR*, were enhanced after PAA treatment. The findings suggest that postharvest application of PAA may be a reliable method to control postharvest decay and preserve quality of harvested pitaya fruit by enhancing the antioxidant potential of the AsA-GSH cycle and activating an antioxidant defense system to alleviate reactive oxygen species (ROS) accumulation.

## 1. Introduction

Pitaya fruit (*Hylocereus undatus*) is a tropical fruit originated from Latin America [1]. According to pulp and peel colour, pitaya fruit is classified into white flesh/yellow peel, white flesh/red peel, and red flesh/red peel fruits [2]. Owing to its desirable taste and texture, and abundant health-promoting compounds, the cultivation and consumption of pitaya have been growing substantially in the recent years [3]. Although pitaya is a non-climacteric fruit, it deteriorates and senesces rapidly after harvest due to the susceptibility to fungal diseases, and physiological disorders leading to shrinkage, thus limiting its storage and marketing potential [4,5]. Several reported treatments, such as cold storage [6], controlled atmosphere [7], plant hormone [8], X-ray irradiation [9] and synthetic chemicals [10], have been proved to control the postharvest diseases and fruit quality deterioration at varying degrees. Nevertheless, there is a continuing search for safer, low-cost, potent senescence inhibitors and antimicrobial technology to maintain quality of harvested pitaya fruit.

Essential oils are now increasingly used for the preservation of several fruits and vegetables due to its safety and antimicrobial properties. *p*-Anisaldehyde (PAA) (4-methoxybenzaldehyde) is a main component of the essential oil derived from seeds of *Pimpinella anisum* [11]. In laboratory media, fruit purees and fruit juices, PAA is confirmed to possess antimicrobial activities against a number of foodborne bacteria, such as *Bacillus subtilis*, *Pseudomonas aeruginosa*, *Listeria monocytogenes*, *Fusarium oxysporum,* and *Staphylococcus aureus*, yeasts (*Candida*) and mold strains (*Aspergillus niger*) [12]. Recently, *p*-Anisaldehyde/*β*-cyclodextrin combination as a fumigation agent effectively suppressed the growth of fungi in strawberry and preserved its storage quality [13]. It implies that PAA might be regulating postharvest physiological and biochemical behaviour of horticultural products. However, the potential of PAA on controlling postharvest deterioration of other postharvest fruits, and its underlying regulatory mechanisms, remains largely unknown.

Postharvest senescence and fruit quality deterioration involve metabolic disorder of reactive oxygen species (ROS) [14]. Overproduction of ROS, including superoxide anion radicals (O_2_^•−^), hydrogen peroxide (H_2_O_2_), and hydroxyl radicals (OH^−^) trigger oxidative damage to macromolecules, resulting in irreversible, deleterious changes in living cells [15]. ROS production is interlinked with ROS scavengers which encompass ROS enzymatic and non-enzymatic systems [16]. Enzymatic scavengers mainly include superoxide dismutase (SOD), peroxidase (POD), catalase (CAT), and the following enzymes involved in ascorbic acid-glutathione (AsA-GSH) cycle: ascorbate peroxidase (APX), glutathione reductase (GR), monodehydroascorbate reductase (MDHAR), and dehydroascorbate reductase (DHAR) [17]. The non-enzymatic scavenger system includes AsA, GSH, α-tocopherol, flavonoids, carotenoids and proline [18]. Mounting evidence from several decades indicates that excessive ROS generation caused by the disruption in the ROS production-scavenging balance can damage the cellular membrane structure, accelerate cell death, and reduce storability of harvested fruits, such as table grape [19], winter jujube [20], blueberries [21], and mango [22]. On the contrary, postharvest treatments, such as near-freezing temperature [23], acidic oxidizing water [24], and essential oils [25] for harvested fruits are proved to retain higher capacity of antioxidant and ROS scavenging ability, which help reduce pathogen infection and maintain fruit quality. In this sense, ROS homeostasis may serve as a common regulatory mechanism for fruits to control senescence process and maintain fruit quality.

Thus, in this work, the changes in physio-chemical properties related to fruit quality, total phenolics and flavones contents, 2,2-diphenyl-1-picrylhydrazyl (DPPH)-free radical scavenging rate, ROS generation, activities of ROS-scavenging enzymes, and components in ASA-GSH cycle in postharvest pitaya that received PAA treatment during storage were investigated. This research aimed to determine the role of ROS metabolism in PAA-mediated maintenance of fruit quality in postharvest pitaya fruit, as well as to validate the effectiveness of PAA treatment as an eco-friendly, safe and promising preservation method for extending the shelf life of harvested pitaya fruits.

## 2. Materials and Methods

### 2.1. Materials and Treatments

Red flesh/red peel pitaya (*Hylocereus polyrhizus* cv. ‘Guanhuahong’) fruits were harvested at the mature stage (~35 d after flower anthesis) from a commerical orchard in Guangzhou, China, and they were transferred to the laboratory immediately. Fruit with uniform shape, colour, size and no physical injuries and disease symptoms were selected and divided randomly into two groups (210 fruits in each group) for the following treatments.

The specific treatment procedures were conducted as follows: (1) PAA treatment- fruits were sprayed with 1 mM PAA solution until the PAA covered the fruit surface uniformly. PAA, at 1 mM, was chosen as the optimum concentration according to a preliminary experiment (data not shown). (2) Fruits evenly sprayed with distilled water served as control group. Thereafter, all treated fruits were air-dried, placed into a plastic box and stored at 20 °C for 15 d with 85–90% relative humidity.

Each treatment comprises three replicates, and samples of 10 pitaya fruits selected from each replicate were taken at Day 0 and at 3-day intervals for assessment of firmness and total soluble solids (TSS). Simultaneously, from the same samples flesh was collected and rapidly frozen at −80 °C for further analysis. For each parameter measurement, there were three replicates in each treatment at each time interval.

### 2.2. Determination of Fruit Physio-Chemical Quality

Every 10 fruits from each replicate were used for decay assessment. Decay incidence was measured based on the spoilage area with a scale composed of 0–5 degrees (0 = absence of decay; 1 =< 10% decay area; 2 = 10–25%; 3 = 25–50%; 4 = 50–75% and 5 => 75%), as described by Liu et al. [10]. The result of decay index was calculated by the equation:

(1) $$\mathrm{Decay}\;\;\mathrm{incidence}\;(\%)\;=\;\frac{\sum (\mathrm{decay}\;\mathrm{scale}\;\;\times \;\;\mathrm{number}\;\mathrm{of}\;\mathrm{fruit}\;\mathrm{in}\;\mathrm{each}\;\mathrm{scale})\;}{\left(5\;\;\times \;\;\mathrm{total}\;\mathrm{number}\;\mathrm{of}\;\mathrm{fruit}\right)}\;\;\times \;\;100$$

Ten pitaya fruits per replication were weighed at Day 0 and at three-day interval during storage period. Weight loss was expressed as a percentage of weight lost compared to the initial weight.

Fruit firmness was measured at three equatorial points of the peeled fruit, using a GY-4 durometer equipment with a cylinder probe (12 mm diameter). The result was expressed as the N. TSS content was assessed by squeezing the fruit from the firmness test onto a digital refractometer (PAL-1, Atago, Japan) and was expressed as a percentage.

Betalain was extracted by homogenizing 0.5 g of sample with 5 mL of 80% methanol (*v/v*) solution by sonication for 10 min, and then centrifuged. Extraction was conducted twice. Betaxanthins and betacyanins were measured using a previously described method [26] through spectrophotometry at wavelengths of 478 nm and 538 nm, respectively. Content of both betalains was expressed as mg 100 g^−^^1^ of fresh weight (FW).

### 2.3. Measurement of Generation Rate of Superoxide Anion Radicals (O_2_^•−^) and Hydrogen Peroxide (H_2_O_2_) Concentration

Production rate of O_2_^•−^ and H_2_O_2_ content in pitaya pulp were determined using a kit (Comin, Suzhou, China), following the procedures of manufacturer’s instructions. NaNO_2_ was used as the standard for calculating the generation rate of O_2_^•−^, which was expressed as nmol g^−^^1^ min^−^^1^ FW. H_2_O_2_ content was calculated with a standard curve constructed by H_2_O_2_, and expressed as μmol g^−^^1^ FW.

### 2.4. Assessment of Activity of Superoxide Dismutase (SOD), Peroxidase (POD), and Catalase (CAT)

Activity of SOD, POD, and CAT was determined using the biochemical kit (Comin, Suzhou, China) following the guidelines of manufacturer. The activity of these enzymes was expressed as unit (U) g^−1^ FW.

### 2.5. Determination of Components in Ascorbic Acid-Glutathione (ASA-GSH) Cycle

The metabolites in the ASA-GSH cycle mainly include AsA, dehydroascorbate (DHA), GSH and oxidized glutathione (GSSG), and their contents were determined according to the methods reported previously [27]. Content of ASA and DHA was calculated using ASA as a standard and were expressed as nmolg^−^^1^ FW. GSH and GSSG contents were calculated based on a standard curve of GSH and GSSG, respectively. The results of GSH and GSSG were expressed as μmol g^−^^1^ FW.

Activity of ascorbate peroxidase (APX), glutathione reductase (GR), monodehydroascorbate reductase (MDHAR), and dehydroascorbate reductase (DHAR) was measured using the reported methods [28]. The activity of all these enzymes was expressed as U g^−^^1^ FW.

### 2.6. Determination of Content of Total Phenolics, Flavonoids, and Scavenging Rate of DPPH Radical

Total phenolics and flavonoids contents were measured in accordance with the procedure as described by Han et al. [29]. The total phenolics content was calculated using the gallic acid as the standard, and result was expressed as mg of gallic acid equivalents (GAE) per gram of fresh weight (mg g^−^^1^ FW). The total flavonoids content was expressed as mg of rutin equivalent per gram of fresh weight (mg g^−^^1^ FW).

The scavenging rate of DPPH radical was determined by a biochemical kit (Comin, Suzhou, China). The absorbance of the reaction system at 515 nm was determined, and the result was finally expressed in percentage terms.

### 2.7. Gene Expression Analyses of Antioxidant Enzymes

Total RNA of pitaya fruit was extracted with EASYspin Plus Plant RNA kit (Aidlab Biotech, Beijing, China), following the manufacturer’s instruction. Hifair™II 1st Strand cDNA Synthesis Super Mix for qPCR and Hieff^®^ qPCR SYBR Green Master Mix (No Rox) (YEASEN Biotech, Shanghai, China) were employed to synthesize cDNA and to preform quantitative real-time PCR (qRT-PCR), respectively. *HpActin1* was selected as the internal control [30]. Gene expression was expressed relative to the expression level of *HpActin*1. The primers used in this study are listed in Supplementary Table S1.

### 2.8. Statistical Analysis

All data presented are means ± standard error of three biological replicates and were subjected to analysis of variance (ANOVA) using SPSS software. Mean values were compared using a Duncan’s test to the significance level (*p* < 0.05 or *p* < 0.01).

## 3. Results

### 3.1. Effects of PAA on Visual Appearance and Physio-Chemical Quality Properties of Pitaya Fruits during Storage

**Figure 1:**
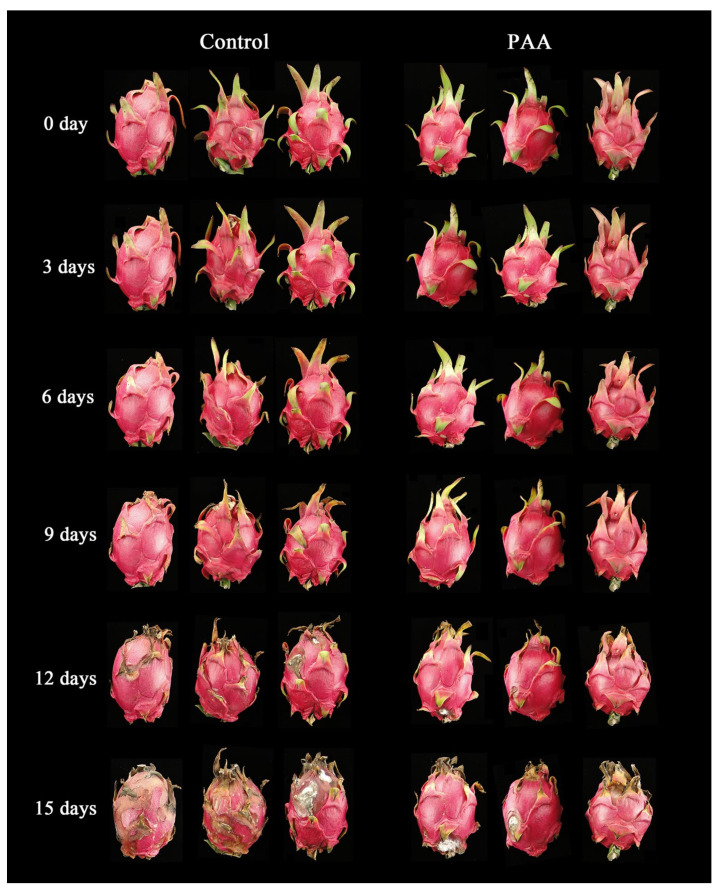
Changes in visual appearance during storage of pitaya fruits treated with PAA.

Visual appearance of pitaya fruits in both groups almost remained unchanged in the initial six days of storage (Figure 1), but shrinkage of bracts and peel, and slight decay symptoms were observed in control fruit on Day 9. Further observations showed that decay, bract degreening, and water loss were more evident in control fruits compared with their counterparts in PAA treatment after 12 d of storage. Comparatively, PAA application maintained better freshness and appearance. On Day 15, the whole fruits decayed extensively in the control, while PAA treatment considerably delayed fruit decay.

**Figure 2:**
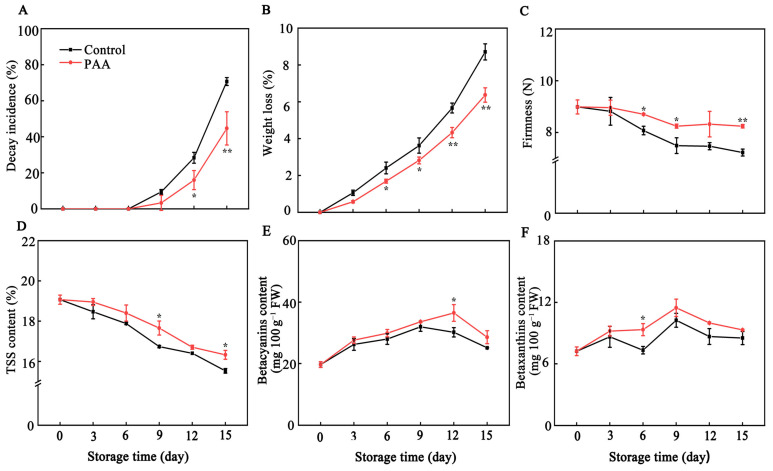
Changes in decay incidence (**A**), weight loss (**B**), firmness (**C**), total soluble solid (TSS) (**D**), betacyanins (**E**), and betaxanthins (**F**) during storage of pitaya fruits treated with PAA. Vertical bars represent the standard error of the mean. The asterisks indicated significant difference between two treatments during the same storage period (* *p* < 0.05, ** *p* < 0.01).

Decay of pitaya fruit was significantly inhibited by PAA treatment (Figure 2A). The decay index was reduced from 70.66% in control to 44.67% in PAA-treated fruit (Figure 2A). Moreover, as shown in Figure 2B, fresh weight decreased throughout storage irrespective of treatment; however, the weight loss in the control group was more pronounced compared to that treated with PAA throughout the experiment. After 15 d of storage, weight loss of PAA-treated fruit was 26.85% lower (*p* < 0.01) than that of control fruit.

Figure 2C showed that fruit firmness of pitaya decreased continuously over the entire storage period. PAA treatment suppressed the loss of firmness during the entire storage. At the final storage time, fruits that were sprayed with PAA still retained firmer (8.24 N) than the control group (7.22 N). For TSS content, regardless of treatment, TSS content of pitaya fruit decreased linearly with storage time. Compared with the initial value (19.07%), TSS content in control pitaya fruits was decreased by 18.56% (*p* < 0.05) at the end of storage, while higher TSS content was observed in PAA-treated fruits throughout storage (Figure 2D).

The contents of betacyanins in postharvest pitaya fruits gradually increased until 9 days after treatment while it decreased over the rest of storage time (Figure 2E). Although no statistically significant differences between two groups were found during the first 9 d, PAA treatment maintained the betacyanins content. On Day 12, the content of betacyanins in pitaya fruits treated with PAA was significantly higher than that of control group, which was 1.14-fold that of control (*p* < 0.05). A similar variation was observed for the betaxanthins content of PAA-treated and control fruit (Figure 2F). Betaxanthin contents in PAA-treated fruit reached the maximum at 11.47 mg 100 g^−^^1^ FW on Day 9, which was 11.90% higher than that in control fruits.

### 3.2. Effects of PAA on Generation Rate of O_2_^•−^ and H_2_O_2_ Concentration of Pitaya Fruits during Storage

**Figure 3:**
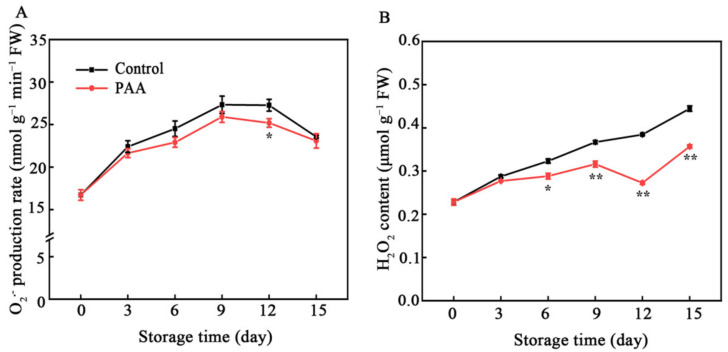
Changes in generation rate of O_2_^•−^ (**A**) and H_2_O_2_ content (**B**) during storage of pitaya fruits treated with PAA. Vertical bars represent the standard error of the mean. The asterisks indicated significant difference between two treatments during the same storage period (* *p* < 0.05, ** *p* < 0.01).

O_2_^•−^ production rate in all both treatments significantly increased from storage Day 1 to Day 9, after which the levels were declined gradually until Day 15 (Figure 3A). However, the generation rate of O_2_^•−^ in PAA-treated fruits was lower than that in control fruits throughout the storage.

H_2_O_2_ content increased in control fruits with storage time (Figure 3B). The accumulation of H_2_O_2_ in control fruits increased from an initial value of 0.23 μmol g^−^^1^ FW to a maximum of 0.44 μmol g^−^^1^ FW after 15 d of storage. PAA treatment significantly inhibited H_2_O_2_ production, in which the concentration of H_2_O_2_ on Day 12 was 19.74% lower than that in control fruits (*p* < 0.01).

### 3.3. Effects of PAA on POD, SOD and CAT Enzymatic Activity and Gene Expression in Pitaya Fruits during Storage

**Figure 4:**
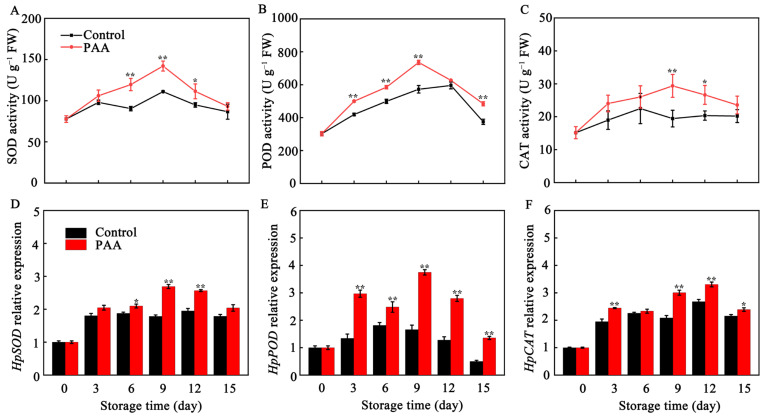
Changes in SOD activity (**A**), POD activity (**B**), CAT activity (**C**), *HpSOD* expression (**D**), *HpPOD* expression (**E**) and *HpCAT* expression (**F**) during storage of pitaya fruits treated with PAA. Vertical bars represent the standard error of the mean. The asterisks indicated significant difference between two treatments during the same storage period (* *p* < 0.05, ** *p* < 0.01).

Activity of SOD and POD exhibited a similar trend, which rose considerably increase at early storage and dropped at the late storage period (Figure 4A,B). The maximum values of POD activity in PAA-treated fruits, and the SOD activity in both control and PAA treatment were all found on the third day. However, control group had the highest level on the twelfth day. Moreover, PAA treatment improved the activity of SOD and POD, with 28.00% and 28.53% higher (*p* < 0.01) than those of control pitaya fruits on Day 9, respectively. CAT activity in control fruits stayed at a stable low level during the whole storage. Until Day 15, CAT activity in PAA-treated fruits was 1.16 times than it was in control (Figure 4C).

As depicted in Figure 4D–F, there was a similar tendency between SOD, POD and CAT enzymatic activity and gene expression. The expression of *HpSOD*, *HpPOD* and *HpCAT* was obviously enhanced by PAA treatment, and a significant difference was found in the expression level of *HpPOD* throughout the storage.

### 3.4. Effects of PAA on Metabolite Content in ASA-GSH Cycle of Pitaya Fruits during Storage

**Figure 5:**
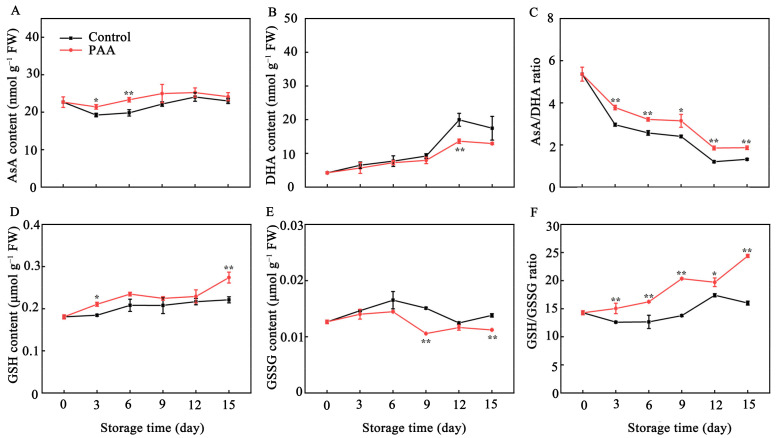
Changes in AsA content (**A**), DHA content (**B**), ratio of AsA/DHA (**C**), GSH content (**D**), GSSG content (**E**) and ratio of GSH/GSSG (**F**) during storage of pitaya fruits treated with PAA. Vertical bars represent the standard error of the mean. The asterisks indicated significant difference between two treatments during the same storage period (* *p* < 0.05, ** *p* < 0.01).

As shown in Figure 5A,B, as storage time progressed, the contents of AsA and DHA in postharvest pitaya fruits peaked on Days 9 and 12, respectively, and then declined. AsA content in fruits treated with PAA was significantly higher than that of control fruits, however, DHA content in PAA-treated pitaya fruits was lower than that of control during the entire storage period. Application of PAA improved the ratio of AsA/DHA in pitaya fruits (Figure 5C). The ratio of AsA/DHA in PAA-treated pitaya fruits was 27.56% and 41.71% higher (*p* < 0.01) than that of control fruits on third and fifteenth day, respectively.

A gradual increase in GSH content was observed in both PAA-treated and control fruits (Figure 5D). Though values of GSH content in both groups showed no difference from storage Day 3 to Day 12, a higher level of GSH content was recorded in PAA-treated fruits during the whole storage. GSSG contents between PAA treatment and control group followed a similar trend, which increased slightly in the early storage period and declined afterwards (Figure 5E). The content of GSSG in PAA-treated pitaya was significantly lower than that in the control fruits on Days 9 and 15. Furthermore, the ratio of GSH/GSSG in pitaya was remarkably increased by PAA treatment compared with control (Figure 5F).

### 3.5. Effects of PAA on the Activity and Gene Expression of AsA-GSH Pathway Related Enzymes in Pitaya Fruits during Storage

**Figure 6:**
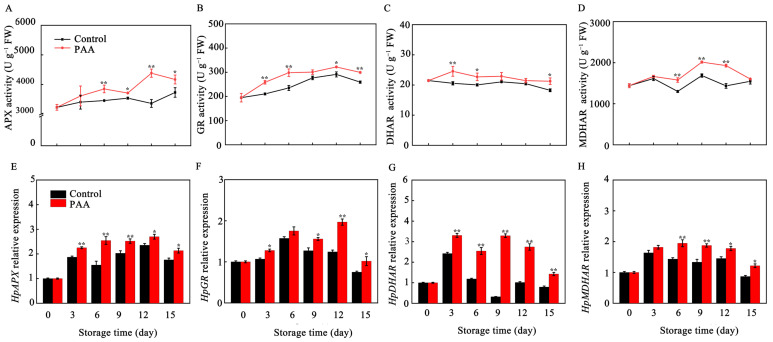
Changes in APX activity (**A**), GR activity (**B**), DHAR activity (**C**), MDHAR activity (**D**), *HpAPX* expression level (**E**), *HpGR* expression level (**F**), *HpDHAR* expression level (**G**) and *HpMDHAR* expression level (**H**) during storage of pitaya fruits treated with PAA. Vertical bars represent the standard error of the mean. The asterisks indicated significant difference between two treatments during the same storage period (* *p* < 0.05, ** *p* < 0.01).

As shown in Figure 6A, APX activity of pitaya fruits increased within the first 6 d, and fluctuated over the rest of storage, irrespective of treatment. PAA treatment resulted in significant increases in APX activity during most of storage. On Days 6 and 12, APX in PAA-treated fruits was 1.11 and 1.30 times higher (*p* < 0.01) than that of control, respectively. GR activity in both PAA-treated and control fruits increased steadily, and reached the maximum level on Day 12, and then declined for the remainder of storage (Figure 6B), but the rate of decline in PAA treatment during late storage was considerably less pronounced than that in the control. DHAR activity, which was higher at 3 d of storage, tended to decline during storage. However, significant differences (*p* < 0.05) of 13.32% and 17.86% over the controls were found after 6 d and 15 d of storage, respectively (Figure 6C). Furthermore, MDHAR activity fluctuated to a greater extent in pitaya fruits during storage (Figure 6D). In comparison to the control, MDHAR activity in the PAA-treated group was higher during the whole storage period, and the difference was extremely significant at 6 d, 9 d, and 12 d, which was 1.21-, 1.19- and 1.34- fold (*p* < 0.01) of control group, respectively.

The relative gene expression of *HpAPX*, *HpGR*, *HpDHAR*, *HpMDHAR* in postharvest pitaya fruits treated with PAA paralleled to those of corresponding enzymes activities. mRNA levels of *HpAPX* and *HpGR* tended to be up-regulated in the earlier storage period and down-regulated during the late storage period (Figure 6E,F). PAA treatment resulted in significantly higher expression of *HpAPX* and *HpGR* throughout the storage time. Similarly, expression of *HpDHAR* and *HpMDHAR* genes were up-regulated in PAA-treated pitaya fruits in comparison with control fruits (Figure 6G,H).

### 3.6. Effects of PAA on Content of Total Phenolics, Total Flavonoids and DPPH Radical-Scavenging Rate of Pitaya Fruits during Storage

**Figure 7:**
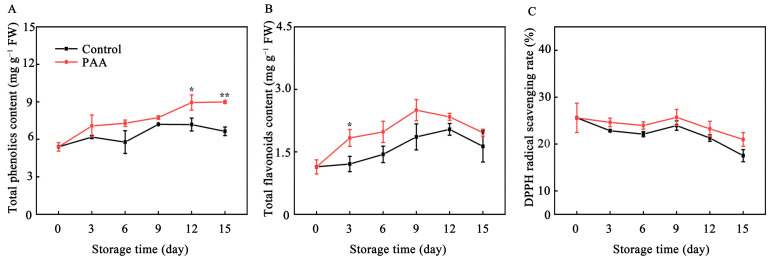
Changes in content of total phenolics (**A**), total flavonoids (**B**) and DPPH radical scavenging rate (**C**) during storage of pitaya fruits treated with PAA. Vertical bars represent the standard error of the mean. The asterisks indicated significant difference between two treatments during the same storage period (* *p* < 0.05, ** *p* < 0.01).

The total phenolic and flavonoids content in control fruits was lower than that in the PAA-treated fruits over the storage period. Total phenolic in control samples declined from Day 9, whereas pitaya fruits in PAA treatment began to decrease from Day 12. Compared with untreated control, PAA-treated fruits showed 0.35- and 0.2-folds higher total phenolic and flavonoids after 15 d of cold storage, respectively (Figure 7A,B).

DPPH radical scavenging rate in both PAA-treated and control fruits during the experiment was shown in Figure 7C, with a persistent decline, except for values at 6 d. However, this decrease was suppressed by PAA treatment. On Day 15, the DPPH free radical scavenging rate of fruits under PAA treatment was 3.46% higher than that of the control.

## 4. Discussion

Postharvest decay is a main limitation for the commercial value and storage life of pitaya fruit. With antimicrobial and insecticidal activity, essential oils are accepted as a prospective option for controlling postharvest fruit quality and safety [31]. The finding of the current study demonstrates that PAA treatment efficiently reduced the decay incidence of pitaya fruits (Figure 2A), which was consistent with the previous studies indicating that PAA could enhance resistance against disease development caused by green mold and blue mold of citrus fruits [32]. In addition, postharvest pitaya fruits undergo a loss of freshness which is characterized by a decline in bract greenness, increased weight loss, decreased fruit firmness and soluble solids [33]. In the present study, visual changes in skin and bract colour obviously varied between PAA treatment and control groups (Figure 1). Furthermore, the result here exhibited that PAA treatment efficiently reduced the weight loss, and delayed the decline in firmness, and TSS (Figure 2B–D) in pitaya fruits during storage. A little lower weight loss observed in PAA-treated fruit than that in the control fruit is possible due to the fact that the PAA functions as a coating agent on the surface of the pitaya fruit, impedes loss of moisture from the fruit. Virtually, weight loss is reportedly interlinked with respiration rate, thus, it is worth exploring the effect of PAA on fruit respiration in the future. As wilting incurred, the firmness of the fruit decreased during storage, while application of PAA maintained higher firmness, which both inhibited the rate of fruit softening and made the fruit less prone to mechanical and microbial damage. Conversely, Lin et al. reported that the fumigation using free PAA induced the loss of firmness, lightness of the surface color and cause a higher water loss [13]. A possible explanation for such opposite results is the differences in species and/or concentrations. Moreover, as a big reservoir of bioactive phytochemicals, pitaya contains betacyanins with remarkable pharmacological values [34]. In this study, a higher content of betacyanins and betaxanthins was retained in PAA-treated fruit as compared with control fruit (Figure 2E,F), which not only functioned as antioxidants but also contributed to maintenance of visual appearance, as the red colour of pitaya fruit is attributed to betacyanins. Therefore, these results suggest that PAA might suppress tissue decay and maintain the nutritional and flavour qualities of pitaya fruits.

According to the available reports, oxidative damage resulting from imbalance in both antioxidant response and ROS-generation affect fruit quality, fruit senescence and resistance to pathogens in most non-climacteric fruits [35]. SOD, POD, and CAT are most studied antioxidant enzymes. SOD dismutates O_2_^•−^ to H_2_O_2_, representing the primary line of resistance against ROS. Then, POD and CAT act synergistically to disintegrate H_2_O_2_ into H_2_O and O_2_ [27]. Enhancing activity of antioxidant enzymes and their associated gene expression to modulate cellular redox homeostasis was previously shown to delay senescence and quality deterioration in various fruits. For example, Chen et al. indicated that enhanced activity of SOD, CAT and APX under 1-methylcyclopropene (1-MCP) treatment contributes to eliminating O_2_^•−^ and maintaining the quality of pears [36]. Melatonin-induced fruit senescence inhibition has been shown to involve enhanced SOD, CAT, APX and POD activities [37]. Moreover, in pear up-regulation of *PcSOD* and *PcCAT* as well as enhanced activity of SOD and CAT reduced H_2_O_2_ production, leading to delayed senescence [38]. In the current study, ROS level in pitaya fruits increased as senescence progressed during storage (Figure 3). PAA markedly improved expression of *HpSOD*, *HpPOD* and *HpCAT* (Figure 4D–F) accompanied by increased activity of SOD, POD and CAT (Figure 4A–C) in harvested pitaya fruits during storage, which led to a lower level of O_2_^•−^ and H_2_O_2_ content in PAA-treated pitaya fruits (Figure 3A,B). These findings indicate that the effect of PAA on reducing accumulation of ROS in pitaya fruits was correlated to the enhanced ROS-scavenging ability at both enzymatic and transcript levels, which, in turn, mitigated oxidative damage and the development of decay and senescence.

Out of the antioxidant enzymes, AsA and GSH have the direct capacity of quenching ROS. In addition, GSH participates in regeneration of AsA through AsA-GSH cycle to remove excess ROS [39]. In the AsA-GSH cycle, APX uses AsA as a substrate to catalyze the reduction of H_2_O_2_ to H_2_O with concomitant production of MDHA, but owing to its unstable property, MDHA can dismutate into DHA or is regenerated into AsA through MDHAR, and DHA is further reduced to AsA by DHAR using reducing equivalents from GSH [40]. GR, a relevant component of the AsA-GSH cycle, catalyzes the conversion of GSSG to GSH form, allowing the maintenance of GSH/GSSG ratio [40]. The protective role of AsA and GSH as well as the ratio of AsA/DHA and GSH/GSSG in enhancing oxidant stress tolerance to delay senescence and maintain quality has been reported in several horticulture products [38,41,42]. Furthermore, given the importance of AsA-GSH cycle in antioxidant and stress resistance, key enzymes and genes involved in this cycle have also been extensively studied. Recently, Zhang et al. reported that 1-MCP induced expression of *AdAPX*, *AdDHAR* and *AdGR* but inhibited two isoforms of *AdMDHAR* expression, which was conducive to elevating the AsA content, scavenging H_2_O_2_, and postponing the senescence of kiwifruit [41]. A similar result was also found in bell pepper, where enhanced AsA-GSH cycle reduced H_2_O_2_, and O_2_^•−^ content, overcoming the physiological disorders during cold storage [43]. Comparably, in our present study, the transcription of *HpAPX*, *HpGR*, *HpDHAR*, *HpMDHAR* was boosted by PAA treatment (Figure 6E–H), which was consistent with the PAA-enhanced activity of APX, GR, DHAR, and MDHAR (Figure 6A–D). Additionally, these enzymes together with higher ratio of AsA/DHA and GSH/GSSG, and lower DHA and GSSG contents (Figure 5A–F) explained the observed lower production of ROS under PAA treatment (Figure 3). These collectively indicate that the increased antioxidant capacity following PAA treatment, resulting in higher content of metabolites, enzyme activity and transcript abundance of genes involved in AsA-GSH cycle, play a vital role in ROS detoxification and redox state maintenance during postharvest storage of pitaya fruits.

In addition, total phenolics, and flavonoids as non-enzymatic antioxidant also fulfill a crucial role in protecting cells from oxidative damage. In plants, DPPH radical-scavenging capacity is generally used to evaluate the total non-enzymatic antioxidant capacity [44]. It has been reported that increasing DPPH radical-scavenging ability, total phenolics and flavonoids is positively correlated with the reduction of ROS and suppression of oxidative events enhanced in postharvest pitaya fruits treated with diphenyliodonium iodide [45], apple polyphenols [1], and methyl jasmonate [3]. In the present study, the decrease of DPPH radical scavenging rate was delayed by PAA treatment (Figure 7C), which was also accompanied by higher contents of total phenolic and flavonoids compared with control (Figure 7A,B), which partially help activate antioxidant responses and inhibit overproduction of ROS.

## 5. Conclusions

Based on above discussion, it is clear that postharvest application of PAA significantly dampened senescence and tissue decay, and effectively maintained the overall quality index of pitaya fruit. The enhanced postharvest disease resistance and quality preservation by PAA treatment might be associated with the reduction in ROS level and an increase in antioxidant capacity. The data suggest that this was attained through enhanced level of total phenolics, flavonoids, and DPPH radical scavenging, and increased gene expression and activity of SOD, POD and CAT as well as AsA-GSH cycle. The present research may help further elucidate the mechanism underpinning PAA-mediated preservation of postharvest pitaya fruit quality.

## Data Availability

Not applicable.

## Supplementary Materials

The following are available online at www.mdpi.com/article/10.3390/foods10102434/s1, Table S1: primers sequence used for qRT-PCR in this study.

## References

[1] Fan P.H., Huber D.J., Su Z.H., Hu M.J., Gao Z.Y., Li M., Shi X.Q., Zhang Z.K. (2018). Effect ostharvest spray of apple polyphenols on the quality of fresh-cut red pitaya fruit during shelf life. Food Chem..

[2] Grimaldo-Juárez O., Terrazas T., Garcia-Velásquez A., Cruz-Villagas M., Ponce-Medina J.F. (2007). Morphometric analysis of 21 pitahaya (*Hylocereus undatus*) genotypes. J. Prof. Assoc. Cactus Dev..

[3] Li X.A., Li M.L., Wang L., Wang J., Jin P., Zheng Y.H. (2018). Methyl jasmonate primes defense responses against wounding stress and enhances phenolic accumulation in fresh-cut pitaya fruit. Postharvest Biol. Technol..

[4] Bellec F.L., Vaillant F., Imbert E. (2006). Pitahaya (*Hylocereus* spp.): A new fruit crop, a market with a future. Fruits.

[5] Zahid N., Ali A., Siddiqui Y., Maqbool M. (2013). Efficacy of ethanolic extract of propolis in maintaining postharvest quality of dragon fruit during storage. Postharvest Biol. Technol..

[6] De Freitas S.T., Mitcham E.J. (2013). Quality of pitaya fruit (*Hylocereus undatus*) as influenced by storage temperature and packaging. Sci. Agric..

[7] Ho P.L., Tran D.T., Hertog M., Nicola B.M. (2020). Effect of controlled atmosphere storage on the quality attributes and volatile organic compounds profile of dragon fruit (*Hylocereus undatus*). Postharvest Biol. Technol..

[8] Li X.A., Li M.L., Wang J., Wang L., Han C., Jin P., Zheng Y.H. (2018). Methyl jasmonate enhances wound-induced phenolic accumulation in pitaya fruit by regulating sugar content and energy status. Postharvest Biol. Technol..

[9] Wall M.M., Khan S.A. (2008). Postharvest quality of dragon fruit (*Hylocereus* spp.) after X-ray irradiation quarantine treatment. HortScience.

[10] Liu R.L., Gao H.Y., Chen H.J., Fang X.J., Wu W.J. (2019). Synergistic effect of 1-methylcyclopropene and carvacrol on preservation of red pitaya (*Hylocereus polyrhizus*). Food Chem..

[11] Shreaz S., Bhatia R., Khan N., Muralidhar S., Basir S.F., Manzoor N., Khan L.A. (2011). Exposure of Candida to *p*-anisaldehyde inhibits its growth and ergosterol biosynthesis. J. Gen. Appl. Microbiol..

[12] Shi C., Zhao X.C., Meng R.Z., Liu Z.J., Zhang G.N., Guo N. (2017). Synergistic antimicrobial effects of nisin and *p*-Anisaldehyde on *Staphylococcus aureus* in pasteurized milk. LWT-Food Sci. Technol..

[13] Lin Y., Huang R., Sun X.X., Yu X., Xiao Y., Wang L., Hu W.Z., Zhong T. (2021). The *p*-Anisaldehyde/β-cyclodextrin inclusion complexes as fumigation agent for control of postharvest decay and quality of strawberry. Food Control.

[14] Lin Y.F., Chen M.Y., Lin H.T., Hung Y.-C., Lin Y.X., Chen Y.H., Wang H., Shi J. (2017). DNP and ATP induced alteration in disease development of phomopsis longanae Chi-inoculated longan fruit by acting on energy status and reactive oxygen species production-scavenging system. Food Chem..

[15] Mittler R. (2017). ROS are good. Trends Plant Sci..

[16] Choudhary A., Kumar A., Kaur A. (2020). ROS and oxidative burst: Roots in plant development. Plant Divers..

[17] Ma Y.Y., Huang D.D., Chen C.B., Zhu S.H., Gao J.G. (2019). Regulation of ascorbate-glutathione cycle in peaches via nitric oxide treatment during cold storage. Sci. Hortic..

[18] Kapoor D., Singh S., Kumar V., Romero R., Prasad R., Singh J. (2019). Antioxidant enzymes regulation in plants in reference to reactive oxygen species (ROS) and reactive nitrogen species (RNS). Plant Gene.

[19] Zhang Z., Xu J., Chen Y., Wei J., Wu B. (2019). Nitric oxide treatment maintains postharvest quality of table grapes by mitigation of oxidative damage. Postharvest Biol. Technol..

[20] Zhao Y.T., Zhu X., Hou Y.Y., Wang X.Y., Li X.H. (2020). Postharvest nitric oxide treatment delays the senescence of winter jujube (*Zizyphus jujuba* Mill. cv. Dongzao) fruit during cold storage by regulating reactive oxygen species metabolism. Sci. Hortic..

[21] Xu F.X., Wang S.H., Xu J., Liu S.Y., Li G.D. (2016). Effects of combined aqueous chlorine dioxide and UV-C on shelf-life quality of blueberries. Postharvest Biol. Technol..

[22] Wannabussapawich B., Seraypheap K. (2018). Effects of putrescine treatment on the quality attributes and antioxidant activities of ‘Nam Dok Mai No.4’ mango fruit during storage. Sci. Hortic..

[23] Zhao H.D., Liu B.D., Zhang W.L., Cao J.K., Jiang W.B. (2019). Enhancement of quality and antioxidant metabolism of sweet cherry fruit by near-freezing temperature storage. Postharvest Biol. Technol..

[24] Chen Y.H., Hung Y.-C., Chen M.Y., Lin M.S., Lin H.T. (2019). Enhanced storability of blueberries by acidic electrolyzed oxidizing water application may be mediated by regulating ROS metabolism. Food Chem..

[25] Taheri A., Behnamian M., Dezhsetan S., Karimirad R. (2020). Shelf life extension of bell pepper by application of chitosan nanoparticles containing Heracleum persicum fruit essential oil. Postharvest Biol. Technol..

[26] Xie F.F., Hua Q.Z., Chen C.B., Zhang L.L., Chen J.Y., Zhang R., Zhao J.S., Hu G.B., Zhao J.T., Qin Y.H. (2020). Transcriptomics-based identification and characterization of glucosyltransferases involved in betalain biosynthesis in Hylocereus megalanthus. Plant Physiol. Bioch..

[27] Tan X.L., Zhao Y.T., Shan W., Kuang J.F., Lu W.J., Su X.G., Tao N.G., Lakshmanan P., Chen J.Y. (2020). Melatonin delays leaf senescence of postharvest Chinese flowering cabbage through ROS homeostasis. Food Res. Int..

[28] Yun Z., Gao H.J., Chen X., Chen Z.S.Z., Zhang Z.Z.K., Li T.T., Qu H.X., Jiang Y.M. (2021). Effects of hydrogen water treatment on antioxidant system of litchi fruit during the pericarp browning. Food Chem..

[29] Han X.Y., Mao L.C., Wei X.P., Lu W.J. (2017). Stimulatory involvement of abscisic acid in wound suberization of postharvest kiwifruit. Sci. Hortic..

[30] Chen C.B., Wu J.Y., Hua Q.Z., Tel-Zur N., Xie F.F., Zhang Z.K., Chen J.Y., Zhang R., Hu G.B., Zhao J.T. (2019). Identification of reliable reference genes for quantitative real-time PCR normalization in pitaya. Plant Methods.

[31] Bakkali F., Averbeck S., Averbeck D., Idaomar M. (2008). Biological effects of essential oils—A review. Food Chem. Toxicol..

[32] Che J.X., Chen X.M., Ouyang Q.L., Tao N.G. (2020). *p*-Anisaldehyde exerts its antifungal activity against *Penicillium digitatum* and *Penicillium italicum* by disrupting the cell wall integrity and membrane permeability. J. Microbiol. Biotechnol..

[33] Chaemsanit S., Matan N., Matan N. (2018). Effect of peppermint oil on the shelf-life of dragon fruit during storage. Food Control.

[34] Jiang H.T., Zhang W., Li X.X., Shu C., Jiang W.B., Cao J.K. (2021). Nutrition, phytochemical profile, bioactivities and applications in food industry of pitaya (*Hylocereus spp*.) peels: A comprehensive review. Trends Food Sci. Technol..

[35] Tian S.P., Qin G.Z., Li B.Q. (2013). Reactive oxygen species involved in regulating fruit senescence and fungal pathogenicity. Plant Mol. Biol..

[36] Chen Y.H., Lin H.T., Shi J., Zhang S., Lin Y.F., Lin T. (2015). Effects of a feasible 1-methylcyclopropene postharvest treatment on senescence and quality maintenance of harvested Huanghua pears during storage at ambient temperature. LWT-Food Sci. Technol..

[37] Wang S.Y., Shi X.C., Wang R., Wang H.L., Liu F.Q., Laborda P. (2020). Melatonin in fruit production and postharvest preservation: A review. Food Chem..

[38] Li X., Li C.Y., Cheng Y., Hou J.B., Zhu J.H., Ge Y.H. (2020). Postharvest application of acibenzolar-S-methyl delays the senescence of pear fruit by regulating reactive oxygen species and fatty acid metabolism. J. Agric. Food Chem..

[39] Soares C., Carvalho M.E.A., Azevedo R.A., Fidalgo F. (2019). Plants facing oxidative challenges-a little help from the antioxidant networks. Environ. Exp. Bot..

[40] Sharma P., Jha A.B., Dubey R.S., Pessarakli M. (2012). Reactive oxygen species, oxidative damage, and antioxidative defense mechanism in plants under stressful conditions. J. Bot..

[41] Zhang Y., Wang K., Xiao X., Cao S.F., Chen W., Yang Z.F., Shi L.Y. (2021). Effect of 1-MCP on the regulation processes involved in ascorbate metabolism in kiwifruit. Postharvest Biol. Technol..

[42] Li C.Y., Wei M.L., Ge Y.H., Zhao J.R., Chen Y.R., Hou J.B., Chen Y., Cheng J.X., Li J.R. (2019). The role of glucose-6-phosphate dehydrogenase in reactive oxygen species metabolism in apple exocarp induced by acibenzolar-s-methyl. Food Chem..

[43] Yao M.Y., Ge W.Y., Zhou Q., Zhou X., Luo M.L., Zhao Y.B., Wei B.D., Ji S.J. (2021). Exogenous glutathione alleviates chilling injury in postharvest bell pepper by modulating the ascorbate-glutathione (AsA-GSH) cycle. Food Chem..

[44] Zhang D.D., Xu X.F., Zhang Z.K., Jiang G.X., Feng L.Y., Duan X.W., Jiang Y.M. (2018). 6-Benzylaminopurine improves the quality of harvested litchi fruit. Postharvest Biol. Technol..

[45] Li X.A., Li M.L., Han C., Jin P., Zheng Y.H. (2017). Increased temperature elicits higher phenolic accumulation in fresh-cut pitaya fruit. Postharvest Biol. Technol..

