# Supplementary material for: Maintenance of Postharvest Quality and Reactive Oxygen Species Homeostasis of Pitaya Fruit by Essential Oil p-Anisaldehyde Treatment
Source: Foods. 2021 Oct 13;10(10):2434. doi: 10.3390/foods10102434 (PMC8535685; doi:10.3390/foods10102434)
Supplement: Supplementary file 1 [file foods-10-02434-s001.zip › foods-1358474-supplementary.pdf]

Supplementary Data

Table S1. Primers sequence used for qRT-PCR in this study.

Gene name	Primer sequence (5'-3')	Primer sequence (3'-5')
HpPOD	***CCATCCCAAATCGCACTATA***	***TAAAGTACGACTCCTGGCTA***
HpSOD	**GAAGCACCATCAGACTTACA**	**GCCATTGAACTTGATAGCAC**
HpCAT	***ATGGCATGAAATTCCCTGAT***	***GAACATGTGCAGACTTTCAG***
HpAPX	***CTTCAAGGAGCTACTGAGTG***	***AGCAGCATATTTCTCAACCA***
HpGR	***ACGTACTGATCATGGTGAAG***	***GTAACATCACCAATAGCCCA***
HpDHAR	***GAGAAATACCCAGATCCACC***	***TCCTGTTCTTTTCCATCGTT***
HpMDHAR	***GCTTCCAGAACCTTCCATTA***	***TTACAGACAATGCAGAGCTT***
